# Editorial: Green growth: innovations in plant science for biostimulant applications

**DOI:** 10.3389/fpls.2025.1748111

**Published:** 2025-12-15

**Authors:** Giuseppe Mannino, Marino Bañón-Arnao

**Affiliations:** 1 Department of Life Sciences and Systems Biology, Plant Physiology Unit, University of Turin, Turin, Italy; 2 Department of Plant Biology (Plant Physiology), University of Murcia, Murcia, Spain

**Keywords:** abiotic stresses, biofortification, food security, genetic improvement, nutritional quality, plant resilience, sustainable agriculture

In the last few years, plant research has experienced a radical transformation: from investigating individual physiological or biochemical processes, it has moved towards a systemic approach involving genetics, ecology, and sustainability (Mannino, 2025). This holistic perspective stems from the urgent need to reconcile three objectives that are often perceived as conflicting: (i) increasing production (Calia et al., 2025), (ii) conserving natural resources (Wazeer et al., 2024), and (iii) improving the quality of agricultural products (Ocwa et al., 2024). The articles collected here, are examples of this new trajectory, where innovation is measured not only in terms of yield, but also in terms of the balance between plants, environment, and humans.

This Research Topic opens with a study by ElShamey et al., dedicated to the phytochemical complexity of tomatoes. The article highlights how phytochemicals are not mere plant metabolites, but key elements for nutritional quality and human health. Their regulation is the result of a delicate balance between genetics, environment, and ripening, in which transcriptional factors, such as *RIN*, orchestrate the accumulation of lycopene and other bioactive molecules. This perspective paves the way for genetic biofortification strategies that, without compromising the naturalness of the fruit, aim to amplify its functional value (Badiyal et al., 2024). The topic of phytochemical upgrading of food is also found in the work of Gatti et al., which explores the effects of a biostimulant based on algae and yeast extracts on the secondary metabolism of apricot trees. Here, the use of this biostimulant demonstrates the possibility of improving the biosynthesis of bioactive compounds, enhancing not only the nutritional profile but also the uniformity and synchronization of fruit ripening.

The core section of the Research Topic is driven by a common theme: how to balance plant productivity with a reduction in environmental impact. In a global context where dependence on synthetic fertilizers is increasingly unsustainable (Sabina et al., 2025), this topic open up concrete scenarios for a transition to more circular agricultural systems, where nutritional management is based on enhanced physiological mechanisms and not only on chemical inputs. For instance, the study by Xu et al. on *Allium ramosum* shows how the combined application of amino acid fertilizers and algae extracts can profoundly alter the biochemical and aromatic profile of flowers, increasing their antioxidant capacity and post-harvest quality. The effects observed by authors demonstrate the potential of growth promoters to modulate plant physiology in a subtle and targeted manner. Similarly, Atero-Calvo et al. explore the role of three amino acid-based biostimulants in lettuce, demonstrating how these products can optimize nitrogen use efficiency and maintain productivity even under conditions of reduced fertilization. In addition to immediate agronomic benefits, their findings suggest broader implications for low-input horticultural systems, indicating that amino acid-based biostimulants could become key tools for decoupling crop performance from high nitrogen dependence (Ali et al., 2024).

**Figure 1:**
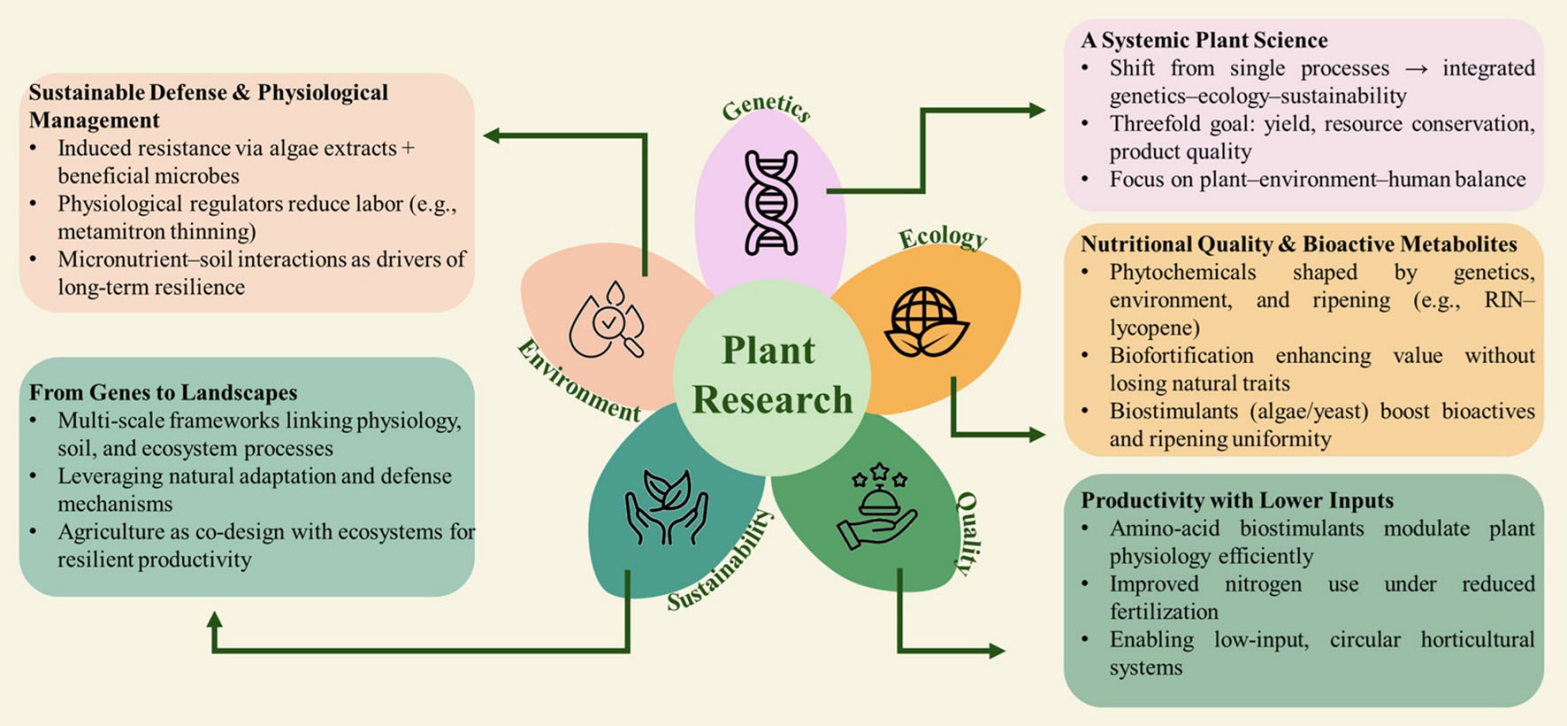
Schematic overview of the main thematic lines addressed by the manuscripts included in this special issue, illustrating the key research areas, conceptual connections, and interdisciplinary perspectives covered by the contributing articles.

The plant’s ability to protect themselves from biotic stress is another pillar of sustainability. The study by Bahmani et al. on grapevines and *Plasmopara viticola* shows how the interaction between an extract of *Ascophyllum nodosum* and the beneficial bacterium *Pseudomonas fluorescens* can synergistically activate the plant’s defense mechanisms. The result is a significant reduction in downy mildew, accompanied by a metabolic and genetic reorganization involving key enzymes in the antioxidant response and signaling hormones such as jasmonic acid. Here, the logic of chemical protection is overturned in favor of induced phytoprotection, which is closer to the rhythms and strategies of nature itself (Mohanta et al., 2025).

While the first studies focus on the molecular and physiological levels, other articles focus to ecological and management processes, completing the multi-scale vision of sustainable agriculture. However, knowledge of plant physiological processes is put to use in more rational and less energy-intensive agronomic management, where fine-tuning of metabolism replaces labor as a lever for optimization (Elazzazy et al., 2025). For instance, Chen et al. analyze the thinning mechanism induced by metamitron in ‘Gala’ apple trees. The substance, acting on photosynthesis and hormonal balance, allows for efficient regulation of fruit set, reducing manual intervention. On the soil side, Wang et al. make an original contribution by exploring the role of available sulfur in the soils of tea plantations at different altitudes. Their analyses reveal an altitudinal sensitivity of the sulfur cycle and its influence on plantation segregation, emphasizing that micronutrients must also be considered in an integrated view of fertility. The study highlights another important physiological aspect, namely that the sustainability of perennial crops cannot be separated from a detailed understanding of the geochemistry of the landscape and its interactions with plant physiology.

Finally, the work of He et al. brings the discussion back to the ecosystem scale, examining how different grazing and resting practices influence the composition of plant life forms and the physical properties of the soil in Tibetan alpine pastures. The results show that near-natural restoration improves soil structure, water retention, and the predominance of hemicryptophytes, promoting the ecological resilience of the system. This study, with its quantitative and systemic approach, ideally closes the circle opened by the initial biochemical research: from the molecule that defends or nourishes, to the landscape that supports plant life in all its complexity.

As these papers are examined together, there is a sense of attending to a narrative that talks not only about genes, enzymes, or soils, but about relationships. Relationships between cells and the environment, between agriculture and landscape, between knowledge and responsibility. Each of these works is a reminder that plants are not objects to be optimized, but living organisms that are in constant dialogue with the world around them, and that ultimately this science is an attempt to learn their language. From greenhouses to Tibetan slopes, a common key-message emerges: plant life has an innate ability to regenerate, to adapt, to transform difficulties into new forms of balance. And a silent, discreet but powerful resilience that should also guide our approach to research. Sustainability, then, is not just a formula to be incorporated into production models, but is a way of looking at living things with respect, of accepting that the most authentic productivity is that which arises from harmony, not control.

## References

[B1] Ali A. Niu G. Masabni J. Ferrante A. Cocetta G. (2024). Integrated nutrient management of fruits, vegetables, and crops through the use of biostimulants, soilless cultivation, and traditional and modern approaches—A mini review. Agriculture 14, 1330. doi: 10.3390/agriculture14081330

[B2] Badiyal A. Mahajan R. Rana R. S. Sood R. Walia A. Rana T. . (2024). Synergizing biotechnology and natural farming: pioneering agricultural sustainability through innovative interventions. Front. Plant Sci. 15, 1280846. doi: 10.3389/fpls.2024.1280846, PMID: 38584951 PMC10995308

[B3] Calia C. García S. G. Ingrao C. Lagioia G. Ruta C. Secchi N. . (2025). Life cycle assessment of microbial plant biostimulant production for application in sustainable agricultural systems. Sci. Total Environ. 981, 179610. doi: 10.1016/j.scitotenv.2025.179610, PMID: 40349559

[B4] Elazzazy A. M. Baeshen M. N. Alasmi K. M. Alqurashi S. I. Desouky S. E. Khattab S. M. R. (2025). Where Biology Meets Engineering: Scaling up microbial nutraceuticals to bridge nutrition, therapeutics, and global impact. Microorganisms 13, 566. doi: 10.3390/microorganisms13030566, PMID: 40142459 PMC11945976

[B5] Mannino G. (2025). Plant-biostimulants interaction: scientific trends, markets dynamics, and real-world implication. J. Plant Interact. 20, 2572668. doi: 10.1080/17429145.2025.2572668

[B6] Mohanta R. Roy S. Ghorai S. Banik S. Lohar A. Thapa S. . (2025). Omics technologies in grapevine stress biology: bridging molecular insights and sustainable viticulture under climate changes. Physiol. Mol. Plant Pathol. 102942. doi: 10.1016/j.pmpp.2025.102942

[B7] Ocwa A. Mohammed S. Mousavi S. M. N. Illés Á. Bojtor C. Ragán P. . (2024). Maize grain yield and quality improvement through biostimulant application: a systematic review. J. Soil Sci. Plant Nutr. 24, 1609–1649. doi: 10.1007/s42729-024-01687-z

[B8] Sabina R. Paul J. Sharma S. Hussain N. (2025). “ Synthetic nitrogen fertilizer pollution: global concerns and sustainable mitigating approaches,” in Agricultural Nutrient Pollution and Climate Change: Challenges and Opportunities (Cham: Springer Nature Switzerland), 57–101.

[B9] Wazeer H. Shridhar Gaonkar S. Doria E. Pagano A. Balestrazzi A. Macovei A. (2024). Plant-based biostimulants for seeds in the context of circular economy and sustainability. Plants 13, 1004. doi: 10.3390/plants13071004, PMID: 38611532 PMC11013454

